# Subcutaneous Versus Submuscular Anterior Transposition of the Ulnar Nerve for Cubital Tunnel Syndrome: A Systematic Review and Meta-Analysis of Randomized Controlled Trials and Observational Studies

**DOI:** 10.1097/MD.0000000000001207

**Published:** 2015-07-24

**Authors:** Chun-Hua Liu, Shi-Qiang Wu, Xiao-Bin Ke, Han-Long Wang, Chang-Xian Chen, Zhan-Long Lai, Zhi-Yong Zhuang, Zhi-Qiang Wu, Qin Lin

**Affiliations:** From the Department of Orthopaedic Surgery, Quanzhou Orthopedic-Traumatological Hospital, Fujian university of Traditional Chinese Medicine (C-HL, X-BK, H-LW, C-XC, Z-LL, Z-YZ, Z-QW); Department of Orthopaedic Surgery, The Second Affiliated Hospital of Fujian Medical University, Quanzhou (S-QW); and Department of Orthopaedic Surgery, Fuzhou Second Hospital of Xiamen University, Xiamen, Fujian province, China (QL).

## Abstract

Subcutaneous and submuscular anterior ulnar nerve transposition have been widely used in patients with cubital tunnel syndrome. However, the reliable evidence in favor of 1 of 2 surgical options on clinical improvement remains controversial.

To maximize the value of the available literature, we performed a systematic review and meta-analysis to compare subcutaneous versus submuscular anterior ulnar nerve transposition in patients with ulnar neuropathy at the elbow.

PubMed, Cochrane Library, and EMBASE databases were searched for randomized and observational studies that compared subcutaneous transposition with submuscular transposition of ulnar nerve for cubital tunnel syndrome. The primary outcome was clinically relevant improvement in function compared to the baseline. Randomized and observational studies were separately analyzed with relative risks (RRs) and 95% confidence intervals (CIs).

Two randomized controlled trials (RCTs) and 7 observational studies, involving 605 patients, were included. Our meta-analysis suggested that no significant differences in the primary outcomes were observed between comparison groups, both in RCT (RR, 1.16; 95% CI 0.68–1.98; *P* = 0.60; I^2^ = 81%) and observational studies (RR, 1.01; 95% CI 0.95–1.08; *P* = 0.69; I^2^ = 0%). These findings were also consistent with all subgroup analyses for observational studies. In the secondary outcomes, the incidence of adverse events was significantly lower in subcutaneous group than in submuscular group (RR, 0.54; 95% CI 0.33–0.87; *P* = 0.01; I^2^ = 0%), whereas subcutaneous transposition failed to reveal more superiority than submuscular transposition in static two-point discrimination (MD, 0.04; 95% CI −0.18–0.25; *P* = 0.74; I^2^ = 0%).

The available evidence is not adequately powered to identify the best anterior ulnar nerve transposition technique for cubital tunnel syndrome on the basis of clinical outcomes, that is, suggests that subcutaneous and submuscular anterior transposition might be equally effective in terms of postoperative clinical improvement. However, differences in clinical outcomes metrics should be noted, and these findings largely rely on the outcomes data from observational studies that are potentially subject to a high risk of selection bias. Therefore, more high-quality and adequately powered RCTs with standardized clinical outcomes metrics are necessary for proper comparison of these techniques.

## INTRODUCTION

Cubital tunnel syndrome, except for carpal tunnel syndrome, is the most commonly nerve compression syndrome of the peripheral nerves.^1–3^ A myriad of surgical approaches have been developed and used since the turn of the century for the treatment of ulnar neuropathy at the elbow, including simple decompression, endoscopic decompression, anterior ulnar nerve transposition (subcutaneous, intramuscular, and submuscular), and medial epicondylectomy.^4–6^

Among them, there is no controversy about the therapeutic effects of subcutaneous and submuscular anterior ulnar nerve transposition for the treatment of cubital tunnel syndrome, which have been widely used to release cubital tunnel, to improve anesthesia or paresthesias and weakness or atrophy of ulnar nerve innervated muscles, and to reduce pain in patients with cubital tunnel syndrome.^1,7,8^ However, it is uncertain whether subcutaneous transposition when compared to submusclar transposition produces better clinical improvement. The reliable evidence in favor of 1 of 2 therapeutic modalities remains controversial.

So far, studies concerning the optimum anterior ulnar nerve transposition technique in the treatment of cubital tunnel syndrome conveyed conflicting results.^9–12^ Unfortunately, due to lack of prospective randomized trials and small sample sizes, these studies were insufficient to determine which anterior ulnar nerve transposition technique is optimum for cubital tunnel syndrome. Thus, in order to pool the reliable and most convincing evidence, we systematically reviewed all available literature reporting clinical outcomes of anterior ulnar nerve transposition and compared the results in series where subcutaneous and submuscular anterior transposition were conducted by meta-analyses. Specifically, we assessed clinically relevant improvement in function compared to baseline, postoperative static two-point discrimination, and adverse events using both surgical modalities.

## MATERIALS AND METHODS

We did not develop a formal protocol for the present one in advance. The present systematic review and meta-analysis were conducted according to the recently published preferred reporting items for systematic reviews and meta-analyses (PRISMA) guidelines.^13^ Additionally, ethical approval or patient consent was not required since the present study was a review of previous published literatures.

### Data Search and Selection Criteria

PubMed, Cochrane Library, and EMBASE databases were searched from inception to February 2015 to identify relevant records reporting the effects of subcutaneous and submuscular anterior transposition of ulnar nerve. We used a combination of search terms related to the type of intervention (“subcutaneous” or “submuscular”) and ulnar neuropathy at the elbow (“cubital/elbow tunnel syndrome” or “ulnar nerve compression/entrapment” or “ulnar nerve sulcus syndrome” or “ulnar neuritis”). No language or publication restriction was imposed. Two investigators (CHL, SQW) independently conducted the initial database search, removed duplicate records, and screened all titles and abstracts for eligibility. Then the full text of the identified articles was reviewed for our inclusion or exclusion criteria. We also manually evaluated the references from relevant retrieved articles and previous systematic reviews or meta-analyses to avoid missing any eligible studies.

Studies were included if it met the following selection criteria: participant – adult patients with primary cubital tunnel syndrome (or ulnar neuropathy at the elbow); intervention – subcutaneous anterior transposition; comparison – submuscular anterior transposition; outcome – clinical outcomes defined as “improved” or “not improved” (at least 12 months of follow-up duration); and design – randomized controlled trials (RCTs) using a truly random or quasi-random allocation and observational studies including retrospective or prospective cohort studies. In addition, we excluded those studies that did not report concrete outcomes data, if no responses were received when we actively contacted the authors to provide further information. Agreements regarding discrepancies between 2 investigators were obtained through discussion.

### Data Extraction and Quality Assessment

Data were independently extracted for first author, year of publication, study location, study type, participant characteristics (intervention, sample size, gender, age, and follow-up data), and outcome data (clinical improvement, static two-point discrimination, and adverse events). Clinically relevant improvement in function compared to baseline was specified as the primary outcome. If more than one clinical outcomes measurement method was evaluated in a study, we selected the better one that was enough to be considered as clinical improvement.

The risk of bias for each RCT was evaluated with the Cochrane Collaboration's Risk of Bias Tool.^14^ The Newcastle–Ottawa Scale was adopted to assess observational studies.^15,16^

### Data Analysis

All meta-analyses were performed using the software Stata/SE 12.0 (StataCorp, College Station, TX) and Review Manager 5.3.5 (Cochrane Collaboration, http://tech.cochrane.org/revman/download). Given wide clinical and methodological differences between RCTs and observational studies,^17–20^ it was decided a priori to analyze the outcomes data separately for RCTs and observational studies. Relative risks (RRs) with 95% confidence intervals (CIs) were computed using the Mantel–Haenszel method for dichotomous outcomes, while standardized mean differences (SMD) with 95% CIs were computed using the inverse-variance method for continuous outcomes. SMD was conducted over weighted mean difference because different measurement indexes that adopted different tools were used in those studies. Heterogeneity across studies was measured by using Cochrane Handbook Q test (*P* < 0.05) and I^2^ statistics.^21^ Random-effect models were applied in the presence of significant heterogeneity (*P* < 0.05, I^2^ > 50%), otherwise fixed-effect models were applied.

To explore possible source of heterogeneity, we further performed subgroup analyses for observational studies based on study of type (retrospective versus prospective cohort studies), setting (single-center versus multicenter), follow-up duration (≤3 years versus >3 years), and region (Asia versus Europe versus North America). We also conducted sensitivity analysis to investigate the influence of each study by omitting a single study sequentially. Publication biases were examined by visually inspecting funnel plot, and the Begg and Egger tests were also performed to evaluate the presence and the effect of publication bias.^22,23^ The *P* values of less than 0.05 were considered as statistically significant. In additional, we incorporated Grading of Recommendations Assessment, Development, and Evaluation (GRADE) approach to evaluate the quality of the evidences for each outcome using a rating system with 4 levels,^24^ which was performed using GRADEprofiler 3.6 (Cochrane Collaboration, http://tech.cochrane.org/revman/other-resources/gradepro/download).

## RESULTS

### Studies Identification and Inclusion

**FIGURE 1:**
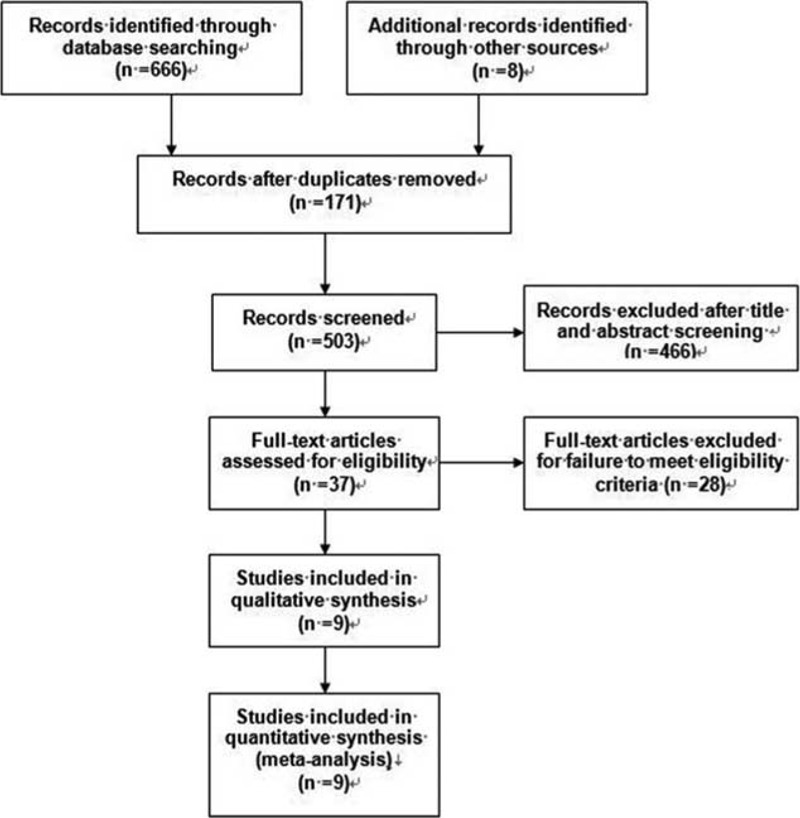
Review flow diagram.

A total of 674 potentially titles and abstracts were identified by the initial electronic database search and other sources. A total of 171 records were removed for duplicates, 41 were excluded as reviews or letter or case report, 414 were excluded as irrelevant to our study, and an additional 11 were excluded as cadaver or animal studies. The remaining 37 full-text articles were obtained and assessed for eligibility, and of these, 28 were further excluded for failure to meet eligibility criteria. Finally, 2 RCTs and 7 observational studies that met the selection criteria were included in our study.^4–6,25–30^ The detail of selection process is listed in Figure 1.

### Study Characteristics

All of the studies included were published from 1984 to 2014. The sample size ranged from 26 to 262 (a total of 356 in subcutaneous group and 249 in submuscular group), and reported average follow-up duration ranged from 1 year to 9.6 ± 3.6 years. Of these studies, 1 was RCT,^29^ 1 was quasi-RCT,^27^ and the other 7 were observational studies (6 retrospective studies^4–6,26,28,30^ and 1 prospective studies).^25^ Among the 9 included studies, 4 were conducted in Asia,^26,28–30^ 4 in Europe,^4–6,27^ and 1 in North America.^25^ One study was multicenter retrospective study.^6^ All studies reported exclusively on comparison of subcutaneous to submuscular anterior transposition of ulnar nerve for the treatment of cubital tunnel syndrome. The detailed information of included studies is shown in Table 1, and outcome data of each included study are summarized in Table 2.

**TABLE 1:**
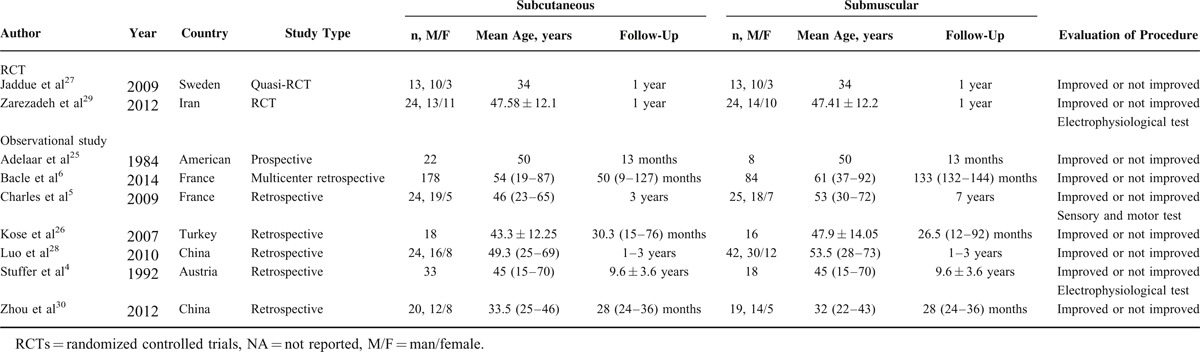
Characteristics of the Included Studies

**TABLE 2:**
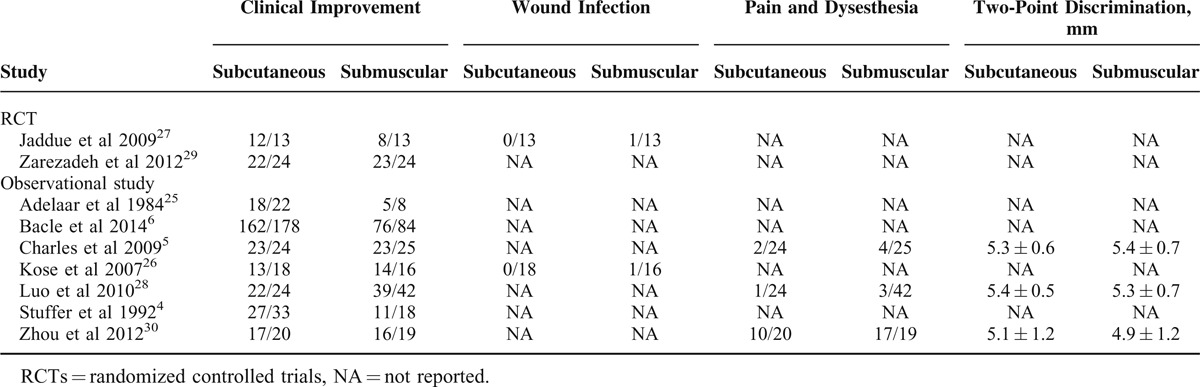
Outcome Data of the Included Studies

### Assessment of Methodological Quality

**FIGURE 2:**
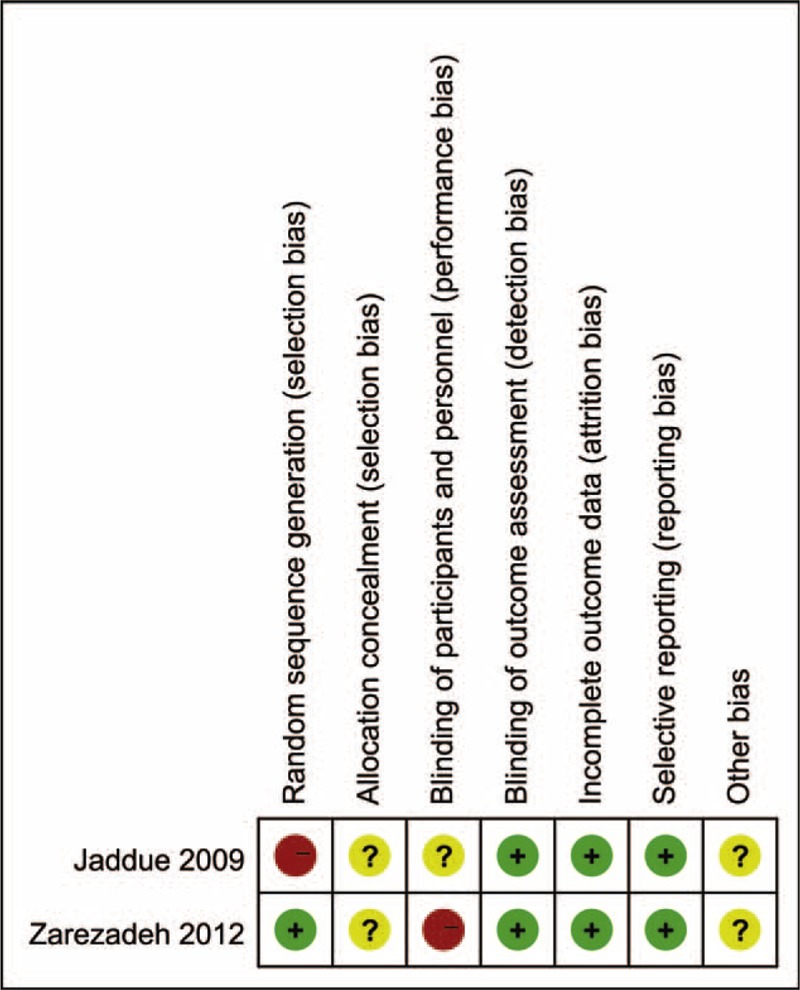
Risk of bias assessment of RCTs: this risk of bias tool incorporates the assessment of randomization (sequence generation and allocation concealment), blinding (participants and outcome assessors), incomplete outcome data, selective outcome reporting, and other risk of bias. The items were judged as “low risk” (+), “unclear risk” (?), or “high risk” (−).

Methodological quality assessment of the 9 included studies is presented in Figure 2 and Table 3. Of the RCTs, Jaddue et al^27^ randomized patients by age (2 years margin) and gender without concrete allocation concealment. Zarezadeh et al^29^ described clearly random sequence generation on the basis of the random table numbers, but there was no adequate method of allocation concealment. Both studies^27,29^ reported all patients were evaluated by the same outcome assessors, and the numbers or reasons for dropout/withdrawal, but unclear blinding of participants and unclear other potential sources of bias. Among observational studies, all studies established comparability between the 2 comparison groups. Scores on the Newcastle–Ottawa Scale, assessing the risk of bias, ranged from 6 to 8 out of 9 indicating a low risk of bias.

**TABLE 3:**
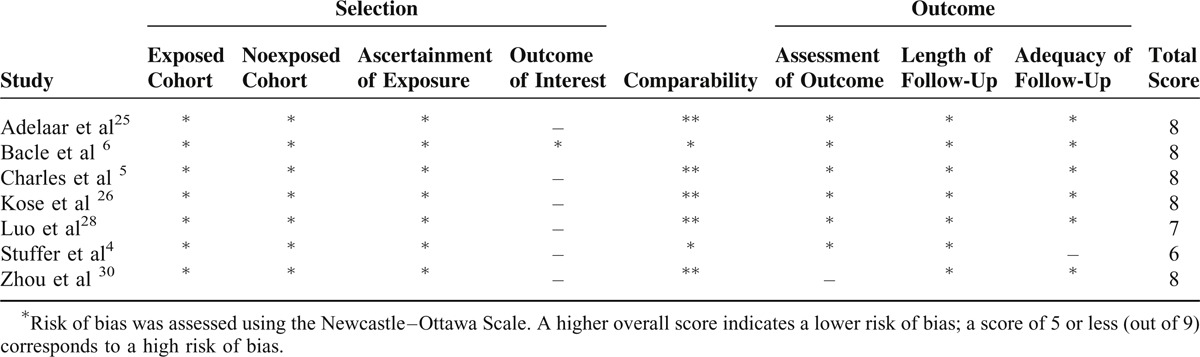
Risk of Bias Assessment of Observational Studies

### Primary Outcome: Clinical Improvement

The primary outcome assessed by the authors for comparison was different among the 9-included studies, leading us to convert it into the dichotomous categories of improved or no improved, as shown in Table 2. Five of the included studies used an established rating system, including Bishop grading system,^27,28^ Wilson–Krout criteria,^26^ the Chinese Medical Society of Hand Surgery Trial upper part of the standard evaluation function assessment,^30^ Visual Analogue Scale,^29^ Yale sensory scale, and ^31^ the Medical Research Council.^32^ In the remaining studies (4/9), the primary end points were measured using author-generated rating system that took into account subjective satisfaction, residual symptoms (evaluated by pain, sensation), and objective parameters (grip strength and static two-point discrimination).

## RESULTS FROM RCTs

**FIGURE 3:**
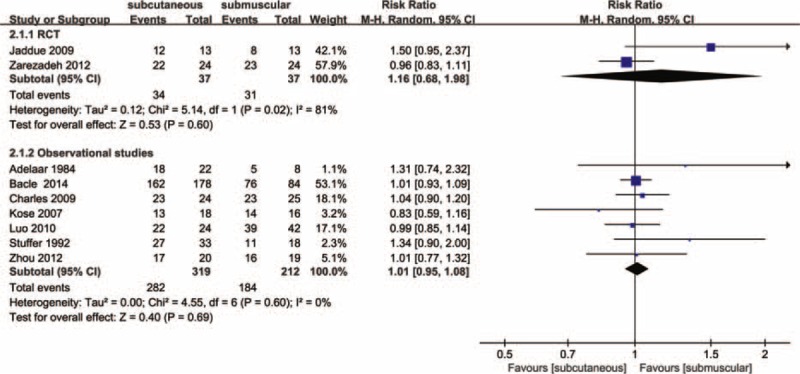
Forest plot of comparison: 1 clinical effect of anterior subcutaneous versus submuscular transposition, outcome: 1.1 proportion of patients with clinical improvement in function compared to baseline.

**FIGURE 4:**
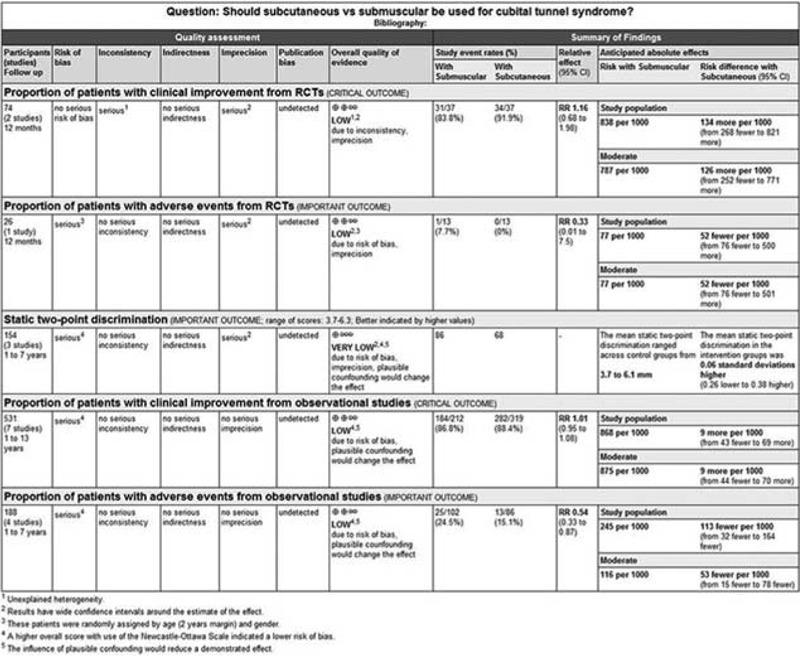
The quality of the evidences for each outcome.

Figure 3 shows the pooled results from the random-effect models with the Mantel–Haenszel method for clinical improvement in function compared to baseline. Overall analysis from 2 RCTs^27,29^ with a total of 74 patients revealed that there was no significant difference between subcutaneous group (34/37) and submuscular group (31/37) in terms of clinical improvement (RR, 1.16; 95% CI 0.68–1.98; *P* = 0.60), with significant heterogeneity between studies (I^2^ = 81%, *P* = 0.02). A random-effects model with the Mantel–Haenszel method was applied. Subgroup analyses or sensitivity analyses were not possible due to small numbers of RCTs, and thus we were not able to explain significant heterogeneity. According to the GRADE, the quality of evidence for this outcome is low due to inconsistency and imprecision (Figure 4).

## RESULTS FROM OBSERVATIONAL STUDIES

In our meta-analysis with the 7 included studies involving 531 patients, subcutaneous and submuscular anterior ulnar nerve transposition are equally effective in patients with ulnar neuropathy at the elbow (RR, 1.01; 95% CI 0.95–1.08; *P* = 0.69); heterogeneity was not found among the studies (I^2^ = 0%; *P* = 0.60; Figure 3), so we used the fixed-effect models. We also carried out 4 subgroup analyses according to the type design (retrospective versus prospective cohort studies), setting (single-center versus multicenter), follow-up duration (≤3 years versus >3years), and region (Asia versus Europe versus North America). The results across all subgroup analyses for observational studies, when comparing patients using subcutaneous transposition to those using submuscular transposition, were consistent with the overall estimate, as shown in Table 4. According to the GRADE, The quality of evidence for this outcome is low due to design limitation and the influence of plausible confounding (Figure 4).

**TABLE 4:**
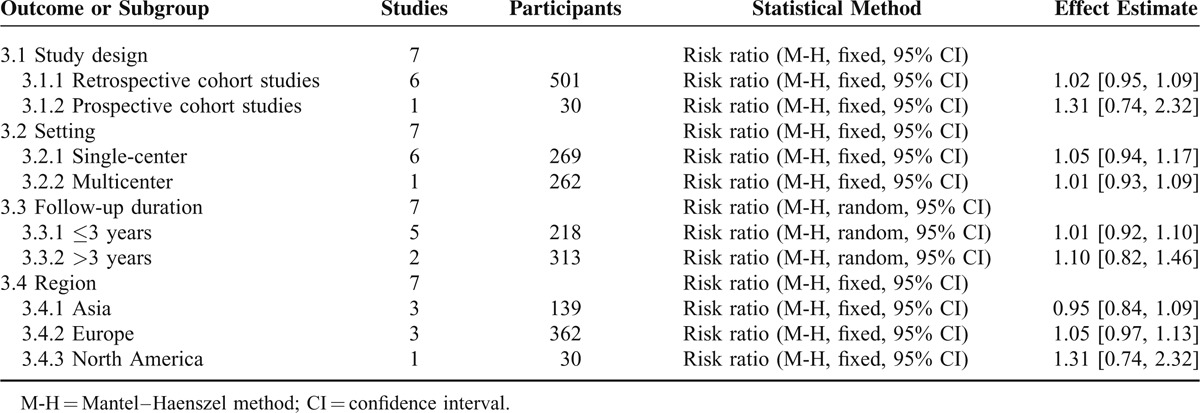
Subgroup Analyses for Clinical Improvement in Observational Studies

## SECONDARY OUTCOMES

### Static Two-Point Discrimination

**FIGURE 5:**
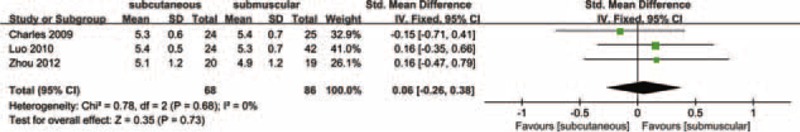
Forest plot of comparison: 2 clinical effect of anterior subcutaneous versus submuscular transposition, outcome: 2.1 static two-point discrimination.

The pre- and postoperative static two-point discrimination was reported in 3 studies^5,28,30^ with a total of 154 patients. All of these studies were observational studies. There was no significant difference in postoperative two-point discrimination between 2 comparison group (SMD, 0.06; 95% CI −0.26–0.38; *P* = 0.73), no heterogeneity was found among the studies (I^2^ = 0%; *P* = 0.68; Figure 5), so we used the fixed-effect models. Further omission of each single study did not substantially alter the overall combined SMD, with a range from −0.01 (95% CI −0.43–0.41) to 0.16 (95% CI −0.23–0.55). Due to design limitation, imprecision, and the influence of plausible confounding, the quality of evidence for this outcome is very low according to the GRADE (Figure 4).

### Adverse Events

Since the included studies rarely dealt with adverse events adequately because the numbers of patients were small, we defined adverse events as complication such as wound infections, pain and dysesthesia of the scar, and worsening of symptoms. Two of the included studies^26,27^ provided data for wound infection (2 in the submuscular group, 0 in the subcutaneous group). Three studies^5,28,30^ described postoperative pain or dysesthesia of the scar, reporting 24 in the submuscular group (n = 86) as compared with 13 in the subcutaneous group (n = 68), but there were no reported patients of worsening of symptoms.

**FIGURE 6:**
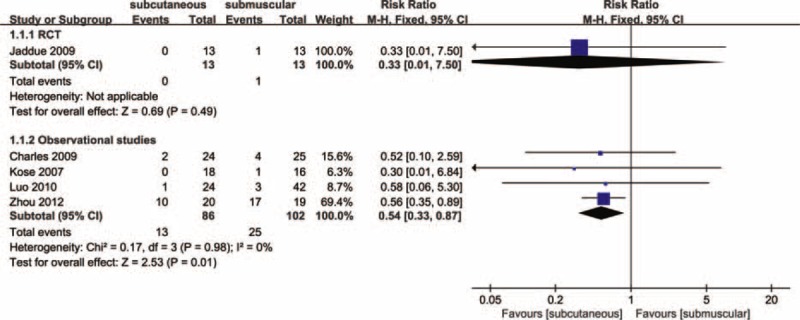
Forest plot of comparison: 3 clinical effect of anterior subcutaneous versus submuscular transposition, outcome: 3.1 proportion of patients with adverse events.

Across the observational studies, 13 postoperative adverse events were identified among the 86 patients who received subcutaneous transposition (15.12 %), compared with 25 among the 102 patients who received submuscular transposition (24.51%). In these studies submuscular transposition increased the risk of adverse events in patients with cubital tunnel syndrome (RR, 0.54; 95% CI 0.33–0.87; *P* = 0.01; I^2^ = 0%; Figure 6). Due to design limitation and the influence of plausible confounding, the quality of evidence for this outcome is low according to the GRADE (Figure 4). In the single RCT^27^ consisting of cubital tunnel syndrome 26 patients, submuscular transposition was associated with a higher number of adverse events (1/13, 7.69%) (1 wound infection in the submuscular group, 0 among the 13 patients in the subcutaneous group). According to the GRADE, the quality of evidence for this outcome is low due to risk of bias and imprecision (Figure 4).

### Publication Bias

**FIGURE 7:**
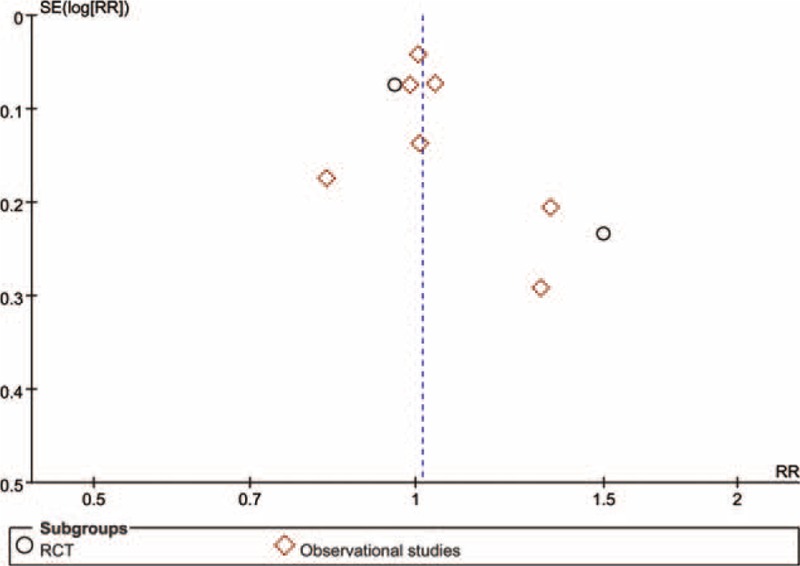
Funnel plot for clinical improvement in patients with cubital tunnel syndrome.

A funnel plot of the included studies that reported clinically relevant improvement in function compared to baseline is shown in Figure 7. Visual inspection of funnel plots showed a slight asymmetry in the lower segments that could be due to insufficient number of studies, potentially leading to a small-study effect. But formal statistical tests indicated the absence of publication bias (Egger test, *P* = 0.251; Begg test, *P* = 0.199).

## DISCUSSIONS

### Main Findings

The present study identified 2 RCTs and 7 observational studies, investigating the effects of subcutaneous and submuscular anterior ulnar nerve transposition in patients with cubital tunnel syndrome. Our meta-analysis showed that no significant differences in the primary outcomes were observed between the 2 comparison groups, both in RCTs and observational studies. These findings were also in accordance with all subgroup analyses for observational studies and our meta-analysis of static two-point discrimination. However, our meta-analysis of adverse events yielded a different result, which the incidence of adverse events was significantly lower in subcutaneous group than in submuscular group. This may draw our attention to the need for further high-quality and adequately powered studies with standardized clinical outcomes metrics.

### Comparison With Previous Studies

To the best of our knowledge, this is the first systematic review and meta-analysis to compare the effects of subcutaneous and submuscular anterior ulnar nerve transposition for the treatment of cubital tunnel syndrome, with moderate-quality RCTs and observational studies. Most previous meta-analyses^33–36^ have focused on the comparison of simple decompression and anterior ulnar nerve transposition (subcutaneous or submuscular). A meta-analysis published in 2000 by Mowlavi et al,^37^ which offered a comparison of 5 techniques (nonoperative treatment, decompression, medial epicondylectomy, subcutaneous, and submuscular transposition), found that subcutaneous transposition produced a high rate (95%) of satisfaction but a low rate (9%) of total relief for minimum-staged patients, whereas submuscular transposition produced the highest rate of satisfaction and total relief (*P* = 0.001) for moderate-staged patients. However, the present meta-analysis suggested that there is no significant difference in the primary outcomes between the 2 treatment modalities. Moreover, all subgroup analyses did not substantially alter our main findings for observational studies, which tested the stability and strength of pooled results.

Similarly, in 2 other meta-analyses^34,36^ focusing on the effects of simple decompression versus anterior ulnar nerve transposition, both RCTs and observational studies were included, but Macadam et al^34^ and Chen et al^36^ were not expected to analyze results separately. According to Cochrane Handbook for Systematic Reviews of Interventions, if RCTs produce significantly smaller or largely effect size than observational studies, results of different study designs should not be combined in a meta-analysis. It may be improper to directly increase heterogeneity. In contrast, the outcomes data in our meta-analysis were analyzed separately for RCTs and observational studies.

Importantly, the majority of previous systematic reviews and meta-analyses analyzed the clinical outcomes as dichotomous outcomes, but Zlowodzki et al^33^ defined the clinical scores as continuous outcomes with use of SMD. To limit the potential source of bias when measuring the primary outcome between different studies, we reviewed the individual studies included in the present study for the number of patients who improved or did not improve with each surgical option, and tabulated clinical improvement to compare 2 surgical treatments, whereas we defined the postoperative static two-point discrimination as continuous outcomes and used SMD.

Consistent with Caliandro et al's meta-analysis,^35^ we excluded an RCT^38^ which compared 2 groups of patients treated by subcutaneous and submuscular ulnar nerve transposition. In this study, the preoperative data of neurophysiological parameters and of cross-sectional area were very similar between comparison groups, which is statistically improbable and a potentially methodological problem. A similar comparison conducted by Lee^39^ demonstrated that submuscular transposition comparing to subcutaneous transposition displayed less perineural scar tissue and healthier axons. But we did not include this study because it was based on the histological study using the rat model. Recently, an observational study^2^ introduced therapeutic modalities including simple decompression, endoscopic decompression, subcutaneous, and submuscular anterior transposition. We also excluded this study^2^ that did not report concrete outcomes data, which no responses were received when we sent an e-mail to the authors for the original raw data.

### Strengths and Limitations of the Study

The present study is a comprehensive evaluation of current evidence, incorporating randomized and observational studies, to compare the efficacy of subcutaneous and submuscular anterior ulnar nerve transposition for the treatment of cubital tunnel syndrome in 1 report. As far as we know, this systematic review and meta-analysis serves as the first attempt to explore the effects of the 2 therapeutic modalities with RCTs and observational studies. We made our best effort to extract all available data from included studies and contacted the authors to provide further information, especially those without concrete outcomes data. We examined the evidence from RCTs and observational studies, and incorporated GRADE approach to summarize evidence to make judgments about the overall quality for each outcome. To evaluate possible source of heterogeneity, we did predefined subgroup analyses only for observational studies due to small numbers of RCTs. In addition, we also performed sensitivity analysis to assess the influence of each study on the overall pooled estimate. Nevertheless, some potential limitations in the present study should also be noted when interpreting the results.

First, the major limitations are only a small number of prospective studies directly comparing 2 surgical treatments and largely depend on retrospectively collected data, which are potentially subject to a high risk of selection bias. Among the 9 included studies, only 2 were small RCTs comprising 74 patients (<2%), whereas the other 7 were observational studies. Moreover, only 4 of them reported that the 2 comparison groups were similar in the baseline characteristics;^26,28–30^ demographic data of the remaining 5 studies were either missing or could not be extracted.^4–6,25,27^ Second, there are no universally standardized metrics to assess clinically relevant improvement in function compared to baseline. Various rating system for improvement exist, but have not been universally adopted. Such lack of a gold standard will result in different definitions of the clinical improvement among studies and may account for a low reliability of the results in our meta-analysis. Therefore, the observed heterogeneity in the primary outcomes from RCTs was likely due to the difference of measurement methods used. Third, there are also differences in operative technique that can influence postoperative clinical curative effects. Variation in operation skills, incision length, operation time, the use of subcutaneous or submuscular anterior ulnar nerve transposition may all have an influence on the clinical effects. The influence of each of these variables on clinical improvement is unknown, and the differences in the incidence of adverse events may be attributable to incision length and operative methods of anterior ulnar nerve transposition.

## CONCLUSIONS

### Implications for Practice

The present study suggests that subcutaneous and submuscular anterior ulnar nerve transposition techniques for cubital tunnel syndrome might be equally effective in the clinical improvement, whereas the incidence of adverse events was significantly lower in subcutaneous group than in submuscular group. Additionally, long-term follow-up outcome data of 2 comparison groups are not available. In practical terms, we invite orthopedic surgeons to select the surgical treatment from the perspective of patient's values and preferences.

### Implications for Research

The quality of available evidence varied from “very low” to “low” (Figure 4), and our main findings largely rely on the outcomes data from observational studies. Future research in this area should include high-quality RCTs with standardized clinical outcomes metrics to evaluate the effectiveness of 2 surgical treatments. These RCTs should be adequately powered to assess all participant important outcomes utilizing standardized clinical outcomes metrics, use the expertise-based design for surgery for the treatment of cubital tunnel syndrome, and report long-term follow-up outcome data.
